# Chloroplast Genomes of Genus *Tilia*: Comparative Genomics and Molecular Evolution

**DOI:** 10.3389/fgene.2022.925726

**Published:** 2022-07-08

**Authors:** Linjun Yan, Huanli Wang, Xi Huang, Yingchao Li, Yuanhao Yue, Zhongwei Wang, Shijie Tang

**Affiliations:** ^1^ Jiangsu Key Laboratory for the Research and Utilization of Plant Resources, Institute of Botany, Jiangsu Province and Chinese Academy of Sciences, Nanjing Botanical Garden, Memorial Sun Yat-Sen, Nanjing, China; ^2^ State Key Laboratory of Tree Genetics and Breeding, Research Institute of Forestry, Chinese Academy of Forestry, Beijing, China

**Keywords:** Malvaceae, chloroplast genome, hypervariable regions, comparison analysis, phylogenetic analysis

## Abstract

*Tilia* is a complex genus in the family Malvaceae that has high ecological and economical values. Owing to the lack of sufficient distinguishable morphological and molecular characteristics, interspecific relationships in this genus are not clear. Chloroplast (cp) genomes are small, meanwhile most angiosperms usually undergo matrilineal inheritance. Consequently, they can be used in molecular systematics and phylogenetic analyses. Here, we sequenced and assembled cp genomes from *T. endochrysea*, *T. tomentosa*, *T. miqueliana*, *T. americana* and *T. cordata,* and compared them with those of seven previously reported *Tilia* species. Similar gene contents, gene orders and GC contents existed among the 12 cp genomes, which ranged from 162,564 to 162,855 bp and encoded 113 unique genes. Abundant simple sequence repeats (119–127) and dispersed repeats (97–135) were detected in *Tilia* cp genomes*.* In total, 11 hypervariable regions were identified that could be suitable for species identification and phylogenetic studies. A phylogenetic analysis of Malvaceae based on 5 hypervariable genes (*matK + ndhF + rpoB + rpoC2+ycf1*) revealed that all eight subfamilies were monophyletic groups. Additionally, the genus *Tilia* was divided into three groups on the basis of all 521 molecular variation loci. The current study provides valuable insights into the genomic evolution of the genus *Tilia*.

## Introduction

Malvaceae, the most diverse family within Malvales, consists of 244 genera and 4,225 species. It is divided into nine subfamilies by phenotypic traits, which are Brownlowioideae, Bombacoideae, Byttnerioideae, Dombeyoideae, Grewioideae, Helicteroideae, Malvoideae, Tilioideae and Sterculioideae. *Tilia* L. (linden, basswood or lime-tree) is a genus of the subfamily Tiliodeae of family Malvaceae, which is widely distributed across the Northern Hemisphere. It has a unique trait that is the bracts adnate to the peduncle of inflorescence, which is intermittently distributed in the temperate and subtropical regions of East Asia and North America, as well as from Europe to western Siberia (Pigott, 2012; Bennett and Alarcón, 2015). *Tilia* are valuable woody species that have long been cultivated around the world, because they are a source of honey, materials for furniture and attractive landscaping trees. *Tilia* also provides good medicinal effects, with its inflorescences and bracts being used as a medicine to treat coughing, anxiety and insomnia-related disorders (Negri et al., 2013; Delnavazi et al., 2015). In addition, higher polyene fatty acids found in the fruit of *Tilia* are considered bioactive compounds (Siger et al., 2021).

In traditional taxonomy, they are usually classified using fruit and leaf characteristics, geographical distribution and cytological features (number of chromosomes) (Mabberley, 2017). Some scholars divided the genus *Tilia* into three groups that is Sect. *Trichophilyra*, Sect. *Lindnera* and Sect. *Tilia* according to a more stable traits, whether the outer pericarp of the fruit is dehiscence or not (Tang and Zhuge, 1996). However, physical traits vary and cross between species, so their relationships and evolutionary histories of *Tilia* species, cannot be adequately differentiated. *Tilia* conservation and taxonomy revision are thus challenging. As the cost of high throughput sequencing was still going down, a well-supported phylogenetic framework of Malvaceae was built based on the whole cp genomes and the nine subfamilies were retrieved, which is a valuable genomic resource to further investigate the evolutionary history at lower taxonomic levels. The phylogeny of four *Tilia* species in China was well resolved based on the complete plastid genome sequences, however, the sampled species are too limited to represent the most species in *Tilia* (Cai et al., 2015).

In eukaryotes, the chloroplast (cp) represents the core organelle for photosynthesis and carbon fixation. It was lack of recombination and slow evolution compared to the nuclear genome, so it was a good candidate for resolving phylogenetic and taxonomic discrepancies and developing species barcoding. It has been widely used in many genera and family phylogenetic analyses, such as those of Poaceae (Feng and Gao, 2021a; Feng et al., 2021b), Cucumis (Zhai et al., 2021) and Adenophora (Kim and Cheon, 2021). Taxonomical discrepancies of some Malvaceae plants had been resolving by identifying part of suitable mutational hotpots. (Abdullah et al., 2019; Abdullah et al., 2020). Compared to the complexity of using a whole cp genome, the hypervariable regions in chloroplast genome could be used as polymorphic and robust markers.

In this paper, we selected five *Tilia* species distributed respectively in Asia, Europe and America, which included the main origins of *Tilia*. Whereafter, we sequenced the cp genomes of the above five *Tilia* species and compared them to the reported cp genomes of seven other *Tilia* species. Meanwhile, all the 12 species were belonged to the all three fruit traits based on traditional morphological classification. Our study mainly aimed to: 1) comprehensively analyze the characteristics of *Tilia* cp genome; 2) determine the phylogenetic relationships among the 12 *Tilia* species; and 3) screen the suitable hypervariable genes or regions for species barcoding and phylogenetic analysis.

## Results

### Characteristics of Chloroplast Genomes

**FIGURE 1:**
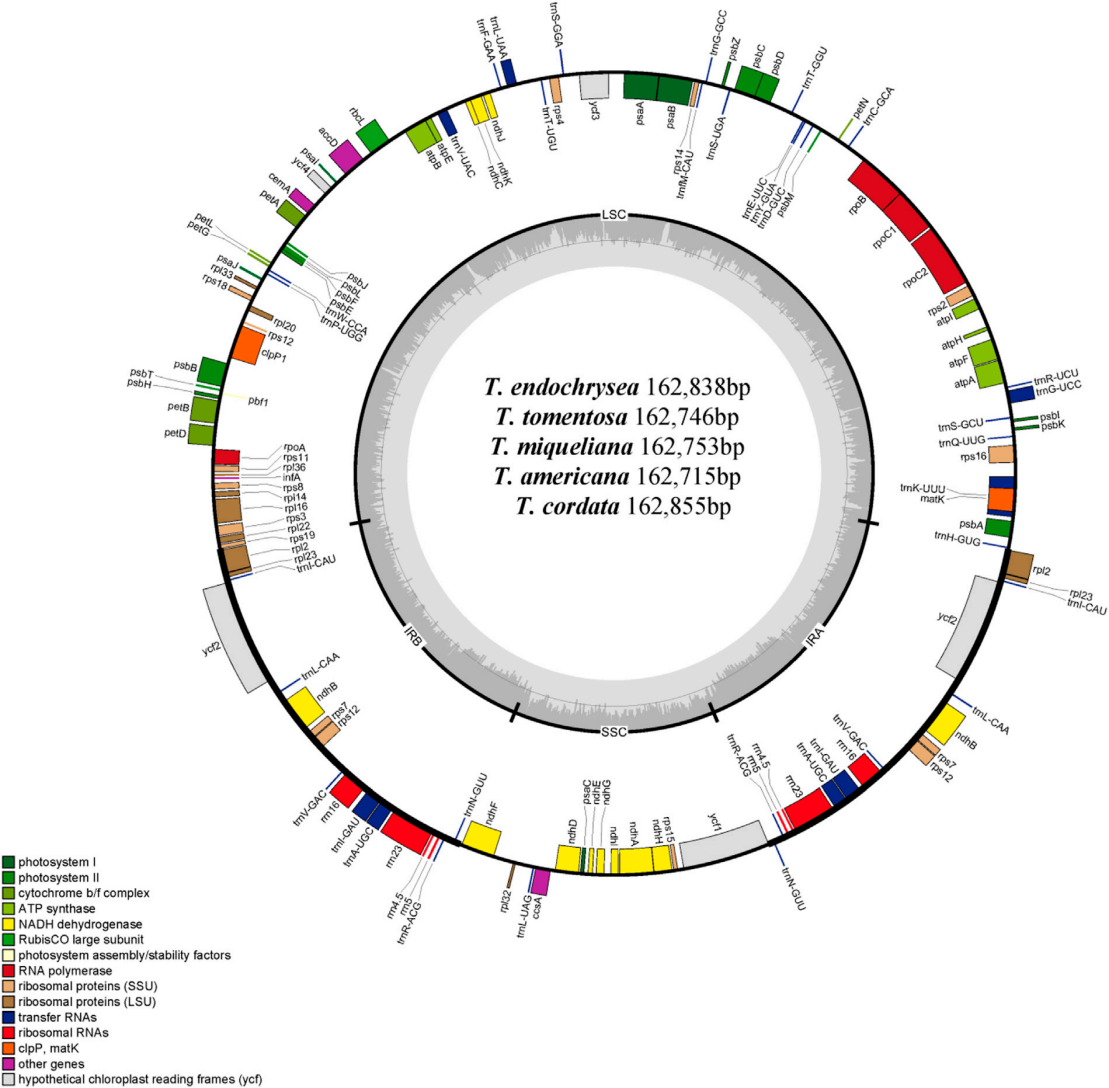
Circular gene map of the chloroplast genomes of *T. endochrysea*, *T. tomentosa*, *T. miqueliana*, *T. americana* and *T. cordata*. Genes drawn within the circle are transcribed clockwise, whereas those drawn outside are transcribed counterclockwise. Genes are color-coded in accordance with their functional groups. The inner circle represents the GC content.

In this study, five cp genome of *Tilia* were sequenced, and 300 M (*T. tomentosa*), 288 M (*T. endochrysea*), 37 M (*T. americana*), 38 M (*T. miqueliana*) and 52 M (*T. cordata*) 150-bp paired-end reads were used for cp genome assemblies. The annotated genome sequences of *T. endochrysea*, *T. tomentosa*, *T. miqueliana*, *T. americana* and *T. cordata* were deposited in GenBank under the accession numbers OK624380, OM908761, OM914582, OM908762 and OM908760, respectively (Figure 1).

Complete cp genome lengths of 12 *Tilia* species ranged from 162,564 to 162,855 bp, and the GC contents were all 36.5%. They exhibited the typical quadripartite structure, consisting of a large single-copy region (LSC, 91,055–91,264 bp, 34.1% GC content), a small single-copy region (SSC, 20,355–20,478 bp, 30.9%–31.0% GC content) and a pair of inverted repeats (IRs, 25,491–25,655 bp, 42.8%–42.9% GC content) (Table 1). All 12 cp genomes contained 130 genes, with 113 being unique, including 79 protein-coding genes, 30 tRNAs and 4 rRNAs. Among them, 17 genes were duplicated in the IR regions, and 18 genes contained a single intron or two introns, including 6 tRNA genes and 12 protein-coding genes (Table 2).

**TABLE 1:** Characteristics of complete chloroplast genomes of *Tilia* species.

Specimens	Length (bp)/GC%	LSC (bp)/GC%	IR (bp)/GC%	SSC (bp)/GC%
*T. endochrysea*	162,838/36.5%	91,264/34.1%	25,580/42.9%	20,414/31.0%
*T. tomentosa*	162,746/36.5%	91,055/34.1%	25,655/42.8%	20,381/31.0%
*T. miqueliana*	162,753/36.5%	91,159/34.1%	25,578/42.9%	20,438/30.9%
*T. americana*	162,715/36.5%	91,205/34.1%	25,571/42.9%	20,368/31.0%
*T. cordata*	162,855/36.5%	91,164/34.1%	25,655/42.8%	20,381/31.0%
*T. mandshurica*	162,796/36.5%	91,127/34.1%	25,649/42.8%	20,371/31.0%
*T. paucicostata*	162,653/36.5%	91,139/34.1%	25,567/42.9%	20,380/31.0%
*T. taishanensis*	162,803/36.5%	91,114/34.1%	25,655/42.8%	20,379/31.0%
*T. oliveri*	162,734/36.5%	91,095/34.1%	25,629/42.9%	20,381/31.0%
*T. insularis*	162,564/36.5%	91,100/34.1%	25,491/42.9%	20,478/31.0%
*T. amurensis*	162,715/36.5%	91,124/34.1%	25,597/42.9%	20,397/31.0%
*T. mongolica*	162,804/36.5%	91,255/34.1%	25,597/42.9%	20,355/31.0%

**TABLE 2:** Genes encoded in the chloroplast genomes of five *Tilia* species.

Category	Gene groups	Gene name
Photosynthesis	Subunits of photosystem I	*psaA*, *psaB*, *psaC*, *psaI*, *psaJ*
	Subunits of photosystem II	*pbf1*, *psbA*, *psbB*, *psbC*, *psbD*, *psbE*, *psbF*, *psbH*, *psbI*, *psbJ*, *psbK*, *psbL*, *psbM*, *psbT*, *psbZ*
	Subunits of NADH dehydrogenase	ndhA*, ndhB*(*×2*), *ndhC*, *ndhD*, *ndhE*, *ndhF*, *ndhG*, *ndhH*, *ndhI*, *ndhJ*, *ndhK*
	Subunits of cytochrome b/f complex	*petA*, petB*, petD*, *petG*, *petL*, *petN*
	Subunits of ATP synthase	*atpA*, *atpB*, *atpE*, atpF*, *atpH*, *atpI*
	Large subunit of Rubisco	*rbcL*
Self-replication	Large subunits of ribosome	*rpl14*, rpl16*, rpl2 *(*×2*), *rpl20*, *rpl22*, *rpl23* (*×2*), *rpl32*, *rpl33*, *rpl36*
	Small subunits of ribosome	*rps11*, rps12**(*×2*), *rps14*, *rps15*, rps16*, *rps18*, *rps19*, *rps2*, *rps3*, *rps4*, *rps7* (*×2*), *rps8*
	DNA-dependent RNA polymerase	*rpoA*, *rpoB*, rpoC1*, *rpoC2*
	Ribosomal RNAs	*rrn16* (*×2*), *rrn23* (*×2*), *rrn4.5* (*×2*), *rrn5* (*×2*)
	Transfer RNAs	trnA-UGC*(*×2*), trnC-GCA, trnD-GUC, trnE-UUC, trnF-GAA, trnG-GCC, trnG-UCC*, trnH-GUG, trnI-GAU*(*×2*), trnK-UUU*, trnL-CAA (*×2*), trnL-CAA, trnL-UAA*, trnL-UAG, trnM-CAU (*×2*), *trnN-GUU* (*×2*), *trnN-GUU, trnP-UGG, trnQ-UUG, trnR-ACG* (*×2*), *trnR-ACG, trnR-UCU, trnS-GCU, trnS-GGA, trnS-UGA, trnT-GGU, trnT-UGU, trnV-GAC, trnV-GAC* (*×2*), trnV-UAC*, trnW-CCA, trnY-GUA, trnfM-CAU
Other genes	Maturase	*matK*
	Protease	clpP1**
	Envelope membrane protein	*cemA*
	Acetyl-CoA carboxylase	*accD*
	C-type cytochrome synthesis gene	*ccsA*
	Translation initiation factor	*infA*
Genes of unknown	Proteins of unknown function	*ycf1*, *ycf2* (*×2*), ycf3**, *ycf4*

*/** genes containing one/two introns. (×2) two gene copies in IRs.

### Simple Sequence Repeats and Dispersed Repeats

**FIGURE 2:**
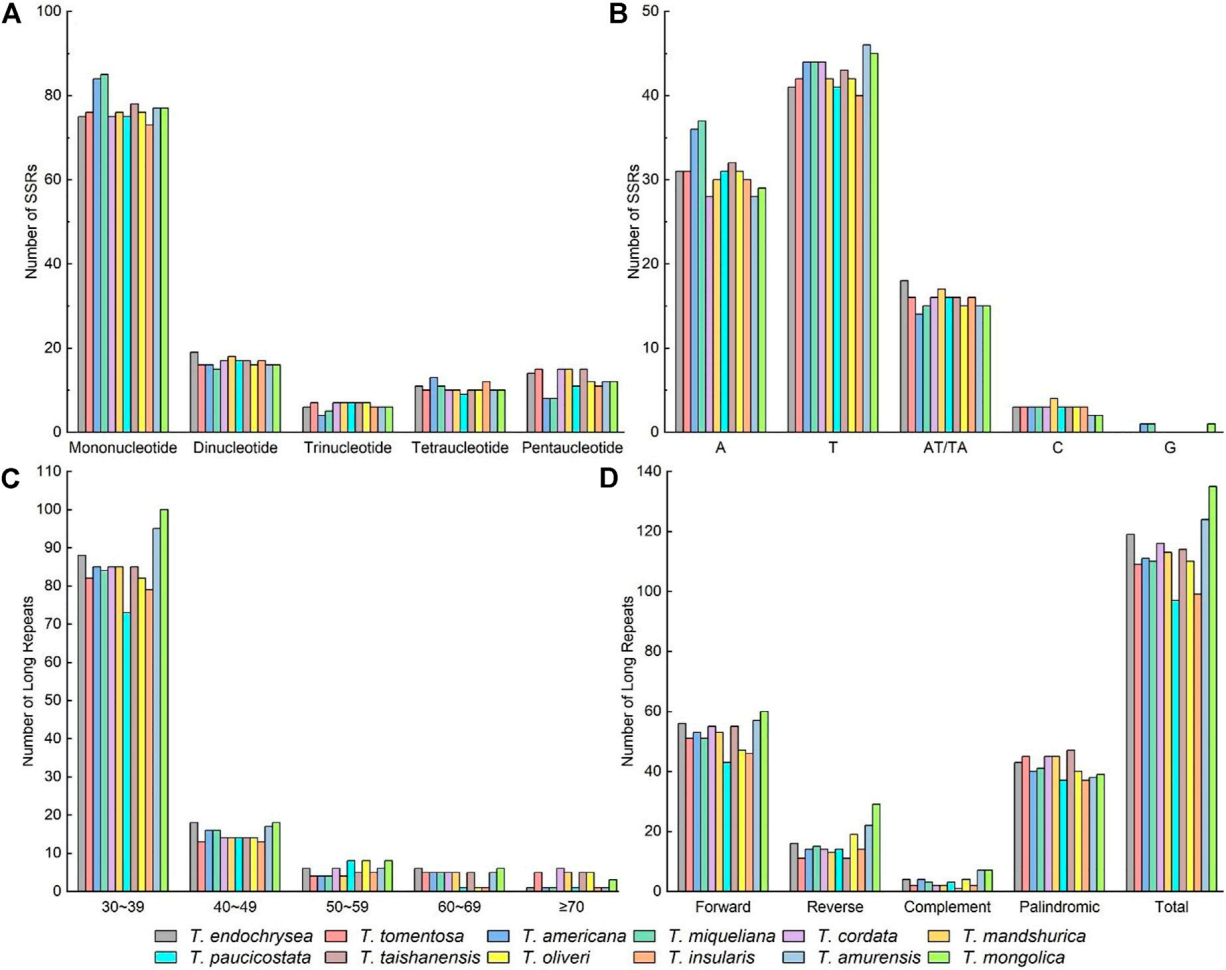
Distribution of SSRs and dispersed repeats in the chloroplast genomes of Tilia. **(A)** Numbers of different SSR types detected in the cp genomes; **(B)** Numbers of different SSR class types; **(C)** Numbers of the four dispersed repeat types in Tilia; **(D)** Numbers of dispersed repeat types having a given length interval (30 to 39, 40 to 49, 50 to 59, 60 to 69 and ≥ 70).

The number of Simple Sequence Repeats (SSRs) in the 12 cp genomes of *Tilia* ranged from 119 to 127. The most abundant SSR type was single nucleotide repeats, accounting for 60.00 %–68.55%, followed by dinucleotide repeats (12.10 %–15.20%), pentanucleotide repeats (6.40%–12.10%), and tetranucleotide repeats (7.56%–10.40%). The trinucleotide repeats were the least common (3.20%–5.88%) (Figure 2A). The A/T-type mononucleotides were the most abundant SSRs, and there were no G-type mononucleotides in the species, except *T. miqueliana*, *T. americana* and *T. mongolica* (Figure 2B)*.* Without taking into account mononucleotide repeats, non-coding regions contained 88.2% of the remaining repeats in the genomes, with the repeats being most abundant in the *rpl33-rps18* and *psbZ-trnG*. Meanwhile, there were abundant SSRs in gene *ycf1* (Supplementary Table S1).

There were 97–135 unique dispersed repeats including forward, complement, reverse and palindromic in the cp genomes of *Tilia* (Figure 2C). Among all the species, forward and palindromic repeats were the most common types, and complement repeats were the least common type. *Tilia amurensis* and *T. mongolica* had the maximum numbers of forward, reverse and complement repeats. Most of the dispersed repeats were less than 40 bp in length, and this was consistent among the 12 species (Figure 2D).

### Inverted Repeat Contraction, Expansion, and Interspecific Comparison

**FIGURE 3:**
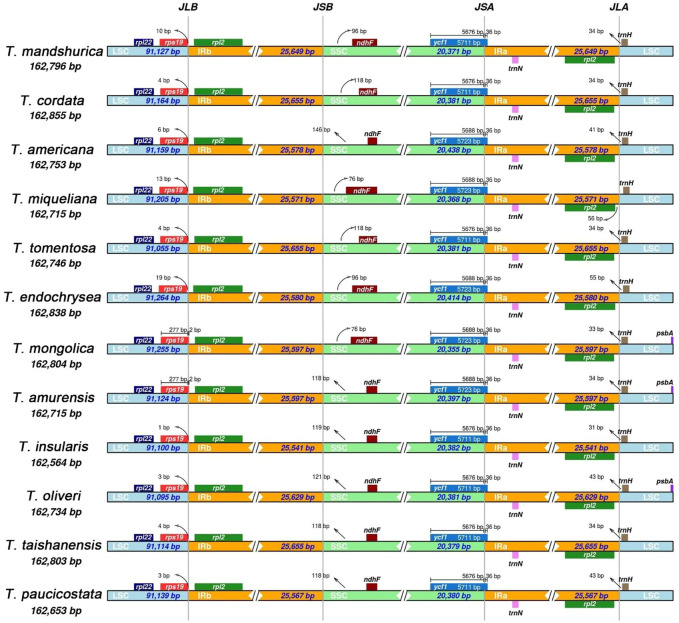
Comparison of the boundaries of large single-copy (LSC), small single-copy (SSC) an inverted repeat (IR) regions of 12 chloroplast genomes of *Tilia*.

We analyzed the junctions of the IRs and the two single-copy regions, along with the placement of adjacent genes in the 12 *Tilia* cp genomes. The genes located at the junctions included *rps19*, *rpl2*, *ndhF*, *ycf1* and *trnH*. The *rps19* and *rpl2* genes were detected at the junction of LSC and IRb. The *rpl2* gene was entirely located within the IRb region. The *rps19* genes of *T. mongolica* and *T. amurensis* spanedspaned the LSC and IRb boundary, unlike in the other 10 *Tilia* cp genomes in which the *rps19* gene was located in LSC region with 1 to 19-bp interval before the LSC and IRb boundary. The *ndhF* genes of 12 *Tilia* are located in the SSC region with 76 to 146-bp intervals to the IRb and SSC boundary. For all 12 species, the *ycf1* genes crossed the SSC and IRa boundary with 36 bp in IRa and the *trnH* gene was located in the LSC region with a 31 to 56-bp interval to the boundary of Ira and LSC (Figure 3).

### Sequence Divergence Analysis

Multiple alignments of 12 *Tilia* plastomes were compared using mVISTA with *T. endochrysea* as a reference. The results revealed a low divergence level among the 12 sequences. In general, coding regions had higher sequence identities than non-coding regions. The main divergences for the coding regions were in *ycf1*, *matK, ndhF* and *rpoC2*, and for the noncoding regions, the most strongly divergent sequences were *psbZ-trnG*, *trnT-trnL*, *atpB-rbcL*, *rpl33-rps18* and *ndhF-rpl32* (Supplementary Figure S1)*.*

**FIGURE 4:**
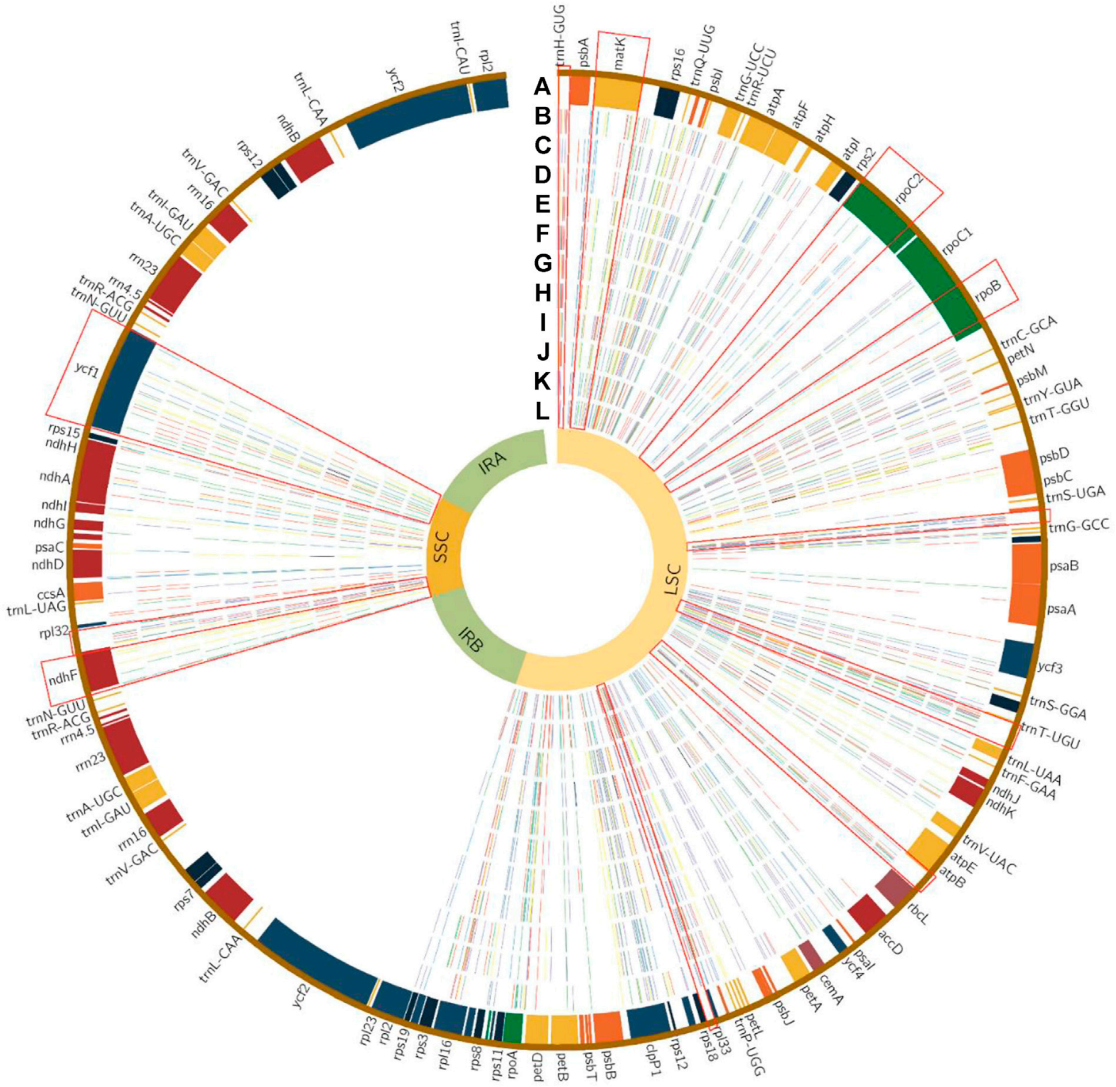
Single nucleotide polymorphic sites in 12 chloroplast genomes of *Tilia.* Track A represents the encoding genes. Tracks B–L represent variant sites in *T. tomentosa*, *T. americana*, *T. miqueliana*, *T. cordata*, *T. mandshurica*, *T. paucicostata*, *T. taishanensis*, *T. oliveri*, *T. insularis*, *T. amurensis* and *T. mongolica* compared with *T. endorchrysea.* The red, blue, yellow and green lines on each track indicate the kinds of variant, A, T, C and G nucleotides, respectively. Purple lines indicate InDel regions. The red rectangles represent the locations of the 11 selected hypervariable regions.

In total, 521 variation sites and 246 InDels were identified in the whole cp genome sequences of the 12 species. Among them, 140 variation sites were in genes, with 59 synonymous variants in 27 genes, 80 missense variants and 79 intron variant. The coding genes having the most variation sites were *ycf1*, *rpoC2* and *rpoB* (34, 12 and 7, respectively). The IGS (intergenic region) having most variation sites were *trnT-trnL*, *ndhF-rpl32*, *psbZ-trnG* and *trnT-psbD*. The coding gene with the most InDels was *ycf1* (Figure 4; Supplementary Table S2)*.*

### Phylogenetic Analysis

**FIGURE 5:**
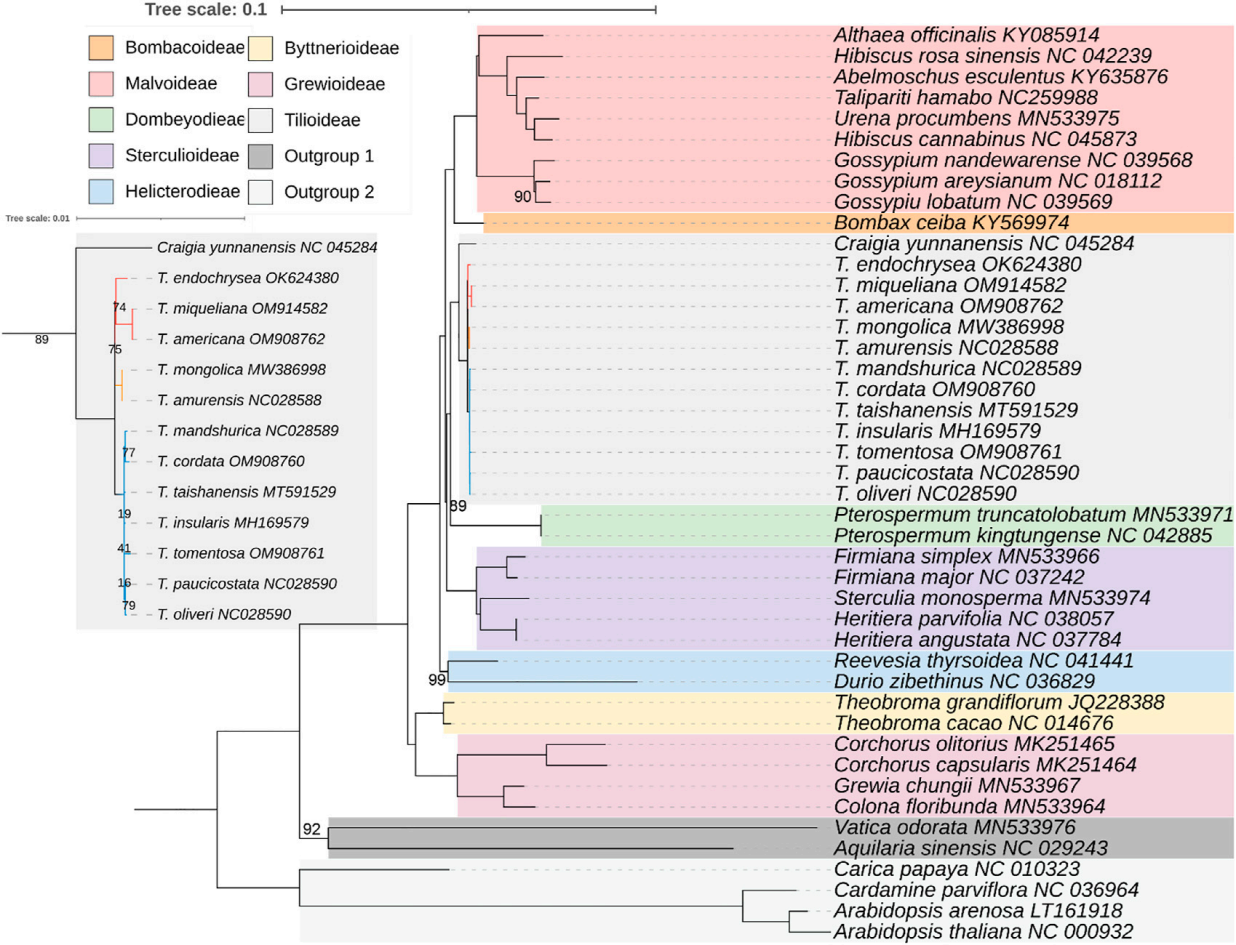
Maximum-likelihood phylogenetic inference of 38 Malvaceae species based on *matK + ndhF + rpoB + rpoC2 + ycf1* genes. The numbers associated with the nodes are bootstrap support and posterior probability values. All the nodes with bootstrapping equal to 100 was not mentioned. 1000 bootstrap replicates.

To explore the phylogenetic positions and evolutionary relationships among *Tilia* species, a maximum-likelihood (ML) tree was constructed using 38 species from 8 subfamilies of Malvaceae. According to the number of mutation loci contained, the maximum five regions were *ycf1*, *matK, nhdF, rpoB*, and *rpoC2.* Five hypervariable region genes were used to construct a phylogenetic tree with two species of Malvales and four species of Brassicales as outgroups, and the variation sites in the 12 *Tilia* species were used to construct a Tilliodeae tree. The phylogenetic tree based on five hypervariable region genes showed *Tilia* was sister to *Craigia* and all eight subfamilies of Malvaceae as monophyletic with strong support. The 12 species of *Tilia* were divided into 3 clades, with *T. americana*, *T. miqueliana* and *T. endochrysea* forming one clade, *T. amurensis* and *T. mongolica* forming one clade, and the remaining 7 species forming the other clade (Figure 5).

**FIGURE 6:**
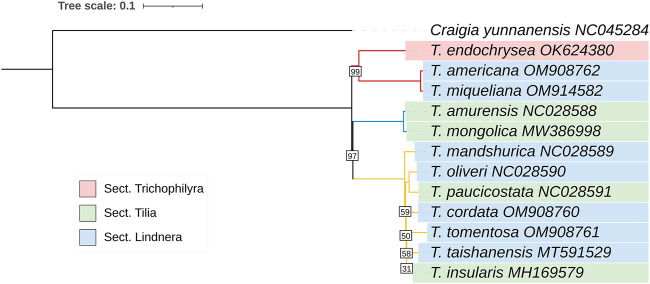
Maximum-likelihood treeof 12 *Tilia* species based on all variation sites. The numbers associated with the nodes are bootstrap support and posterior probability values. All the nodes with bootstrapping equal to 100 was not mentioned. 1000 bootstrap replicates.

In the phylogenetic tree constructed using all variation sites, the 12 *Tilia* species were divided into 3 clades with a bootstrap value of 1,000 and *Craigia yunnanensis* as the outgroup. The three clades were as follows: 1) *T. americana*, *T. miqueliana* and *T. endochrysea*; 2) *T. amurensis* and *T. mongolica*; 3) *T. mandshurica*, *T. cordata*, *T. tomentosa*, *T. insularis*, *T. taishanensis*, *T. paucicostata* and *T. oliveri* (Figure 6)*.*

Combining the data of the two evolutionary trees, the branch lengths leading to *Tilia* were short, indicating that *Tilia* evolved more slowly than other species in Malvaceae.

## Discussion

Compared with the seven previously sequenced cp genomes, the five newly sequenced cp genomes had similar lengths and exhibited the typical quadripartite structure. The number of introns and positions of genes were identical in all 12 species, which indicated that the cp genome was highly conserved during evolution. Overall, the lengths of the 12 *Tilia* cp genomes, ranging from 162,564 to 162,855 bp, were larger than most species of Malvaceae, and the GC contents of the *Tilia* species (36.5%) was at the low level among the Malvaceae (Wang et al., 2021), being slightly lower than *Gossypium* (37.2%–37.4%) (Chen et al., 2016), *Theobroma* (36.9%) (Jansen et al., 2011), *Bombax* (36.8%) (Gao et al., 2018) and *Heritiera* (36.8%) (Zhao et al., 2018). In general, the over-all GC content is an important species indicator (Shen et al., 2017). A high GC content is often associated with the earlier divergence of a phylogenetic position (i.e., Nymphaeales and Magnoliids) (Cai et al., 2006). Therefore, we concluded that *Tilia* differentiated later among the Malvaceae. This conclusion was consistent with divergence time estimation of Malvaceae based on coding gene sequences (Wang et al., 2021).

SSRs often contain 1–6 nucleotides and are primary sources of molecular markers for population genetics and biogeographic studies (Kyalo et al., 2018; Mustafina et al., 2019). In this study it was consistent with previous research results that single nucleotide repeats of A/T were the most common among all the repeat types (Qian et al., 2013; Yi and Kim, 2016; Jiang et al., 2017). A potential reason for the higher frequencies of the A/T repeats is polyadenylation at the end of mRNAs in the cp genes of many species. Additionally, the strand separation for A/Ts is relatively easier than G/Cs during plastome replication, which increases slipped-strand mispairing (Zhao et al., 2020). Meanwhile, SSRs with two to five repeat motifs were found in the 12 cp genomes, with 88.2% of them localized in non-coding areas (88.2% on average). SSRs tend to appear in the cp non-coding regions because of the strong selection against them in coding regions (Menezes et al., 2018). The SSRs identified will be helpful in future population genetic studies and evolutionary analyses. Occasional short sequence repeats and mutations in DNA sequences can be extended into longer tandem repeats through slip-chain mismatch events, and transposon-mediated insertion and copy slip may be responsible for both direct and reverse dispersed repeats (Levinson and Gutman, 1987). In this paper, the forward and palindromic repeats were the most common repeat types and most of the dispersed repeats were less than 40 bp, as the previous studies was reported (Liu et al., 2019; Kirov et al., 2020; Luo et al., 2021).

Although IR regions are highly conserved in most plants, especially within the same genus, structural variations in the IR/SC boundary regions are very common. The contraction and expansion of IR/SC regions are the main reasons for plastid length differences (Zhu et al., 2016; Yu et al., 2019), and they play important roles in evolution (Kim and Lee, 2004; Wang et al., 2008). By comparing boundary regions among the 12 *Tilia* cp genomes, we found that the numbers and orders of genes were conserved (Li et al., 2018; Ye et al., 2019). Previous studies showed that the *rps19* genes in some species of plants (Mu et al., 2018; Xie et al., 2018; Zhang et al., 2020; Li et al., 2021) were pseudogenes, and they were found both in the IRa or the IRa/LSC boundary and in the IRb or the IRb/SSC boundary. However, there is only one copy of *rps19* in the *Tilia* cp genomes. Meanwhile, some differences existed at the boundaries, with the most obvious being for *rps19* in *T. mongolica* and *T. amurensis* located at the boundary of IRa/LSC. It suggested that *T. mongolica* and *T. amurensis* had similar cp gene structures, which were more closely related in evolutionary terms.

As other woody species genera, the 12 whole-cp genome sequences showed low divergence in general. For the *Tilia* tree, it takes 6 to 40 years for beginning to flower and produce seed. The low mutation rates in these cp genomes may be attributed to their long generation times (Ren et al., 2018). *ycf1*, *ndhF*, *rpoB*, *rpoC2*, *matK*, *trnH-psbA*, *psbZ-trnG-*, *trnT-trnL*, *atpB-rbcL*, *rpl33-rps18* and *ndhF-rpl32*. Our results showed that *ycf1*, *ndhF*, *rpoB*, *rpoC2*, *matK*, *trnT-psbD*, *psbZ-trnG*, *trnT-trnL*, *atpB-rbcL*, *rpl33-rps18* and *ndhF-rpl32* were the mutational hotspots, which were partly the same as the other Malvaceae species and many other species (Zhao et al., 2015; Mo et al., 2020; Wang et al., 2021). These regions could be good candidate regions for *Tilia* species identification. In addition, 521 variation sites and 246 InDels were identified in the 12 *Tilia* species. Most of the variants were in the intron and missense mutations, which might be related to evolution under positive selection (Kim et al., 2017). Through the analysis, we found that the most of mutational hotspots were concentrated in IGS, which was consistent with the occurrence area of SSRs. The variation sites overlapped with the abundant areas of SSRs (*ycf1*, *rpl33-rps18* and *psbZ-trnG*). Such a phenomenon has been confirmed in Malvaceae (Abdullah et al., 2021a), and be universal in plant cp genomes. This co-occurrence of subsitutions with repeats supported the repeats could be used as identification of mutational hotpots (Li et al., 2020; Abdullah et al., 2021b).

We selected five hypervariable region genes (*matK, nhdF, rpoB, rpoC2 and ycf1*) for phylogenetic tree construction. All eight subfamilies of Malvaceae were monophyletic with high bootstrap support values, and the genera *Tilia* was divided into three clades. Thus, some genes were shown to act as a promising plastid genomic barcode, such as *ycf1*, *matK* and *rbcL* (Dong et al., 2015; Amar, 2020). Chloroplast genes were highly conserved sequences in general. Compared with full-length genes, it is more reliable and convenient to construct an evolutionary tree with hypervariable genes. In order to improve the bootstrap support values of the phylogeny tree genus *Tilia*, we reconstructed a ML tree of *Tilia* based on all variation sites. It is revealed that the phylogenetic relationship of *Tilia* based on all variation sites was consistent with tree constructed by the hypervariable five genes. The conflicts between molecular and morphological characteristics existed in genus *Tilia*. For example, in traditional taxonomy, genus *Tilia* is divided into three groups based on fruit morphology, Sect. *Trichophilyra*, Sect. *Lindnera* and Sect. *Tilia*. Whereas, Sect. *Trichophilyra* was a paraphyletic group and Sect. *Lindnera* and Sect. *Tilia* are polyphyletic based on molecular evidence. According to the molecular phylogenetic evidence, *T. miqueliana* and *T. americana* were more closely related to *T. endochrysea*. *T. insularis* is a subspecies of *T. amurensis* on basis of the vegetative and reproductive morphological evidence (Pigott, 2012; Yang et al., 2018), but the results of this study suggested that *T. amurensis* and *T. insularis* are two individual species and *T. amurensis* is more closely related to *T. mongolica.* The conflicts between molecular and morphological due to the long reproductive cycles, long history of anthropogenic spread of *Tilia* and recently rapid differentiation by frequently occurred interspecies hybridization (Pigott, 2012; Cvetković et al., 2021).

In this study, we successfully reconstructed the phylogenetic trees of Malvaceae and *Tilia*, and they indicated that plastid phylogenomics could be used to determine the interspecific relationships within the genera *Tilia*. Meanwhile, the hypervariable regions would be selected to build the phylogenetic tree and develop species barcoding.

## Materials and Methods

### DNA Sequencing, Genome Assembly and Gene Annotation

Fresh leaves were obtained from adult trees of *T. endochrysea*, *T. tomentosa* “*Sterling*”*, T. miqueliana*, *T. americana* and *T. cordata* planted at the Institute of Botany, Jiangsu Province and Chinese Academy of Sciences, Nanjing, China. Total genomic DNA was extracted using a modified cetyltrimethylammonium bromide method and applied to 150-bp paired-end library construction using the NEBNext Ultra DNA Library Prep Kit for Illumina (New England BioLabs, Ltd., United States) sequencing. Sequencing was carried out on the Illumina NovaSeq 6000 platform (BIOZERON Co., Ltd., Shanghai, China). *De novo* assembly of the cp genome of the closely related species *T. oliveri* (NC 028590) using NOVOPlasty (Dierckxsens et al., 2017) produced two circular optional contigs of the cp genome. The candidate cp genome was chosen because it had the highest resemblance to *T. oliveri* cpDNA. BLAST searches against cp genomes of the related species *T. oliveri* and the NOVOPlasty results were used to retrieve a number of possible cp reads from the pool of Illumina data. Using SPAdes-3.13.0 software (Bankevich et al., 2012), cp Illumina data were collected to perform cp genome *de novo* assembly. The scaffolds from the SPAdes-3.13.0 (Antipov et al., 2016) result were used to optimize the NOVOPlasty assembly contig, which was then aligned with the original clean Illumina reads using Burrows-Wheeler-Alignment Tool (Li and Durbin, 2009) and base corrected using Pilon v1.22 (Luo et al., 2012). The assembled sequences were then reordered and orientated in accordance with the reference cp genome, resulting in the final assembled cp genomic sequence. The plastid genome annotator tool (Qu et al., 2019) was used to annotate the genomes, and the start and end codon boundaries were manually corrected if necessary.

### Identification of Repeat Sequences and Simple Sequence Repeats

REPuter (Kurtz et al., 2001) was used to identify repeat sequences, which had four forms t: forward, reverse, complementary and palindromic, in the cp genomes. The detection parameters were set to a minimum repeat size of 30 bp and a 3-bp edit distance.

The MicroSatellite identification program (Beier et al., 2017) (https://pgrc.ipk-gatersleben.de/misa/) was used to identify SSRs in the cp genome sequences using the following parameter settings: size of a unit (nucleotide) 1_10, 2_5, 3_4, 4_3, 5_3, 6_3 were the minimum repetitions. A minimum spacing of 100 bp was imposed between two SSRs.

### Comparison of Genome Structures, and an Inverted Repeat Region Contraction and Expansion Analysis

Using IRscope (https://irscope.shinyapps.io/irapp/), the placements of IR, SSC, and LSC junctions were compared in the 12 cp genomes. mVISTA was used to visualize the variations among the 12 *Tilia* cp genomes (default parameters and LAGAN mode) (Frazer et al., 2004).

### Polymorphism Analysis and Phylogenetic Analysis of Chloroplast Genomes

Each sample was compared with *T. endochrysea* as a global reference sequence using snippy software (https://github.com/tseemann/snippy).

The cp genomes of 38 species were downloaded from the National Center for Biotechnology Information for the phylogenetic analysis. A phylogenetic tree based on the *matK + ndhF + rpoB + rpoC2 + ycf1* matrix of the studied species was constructed using *Aquilari sinensis*, *Vatica odorata*, *Carica papaya*, *Arabidopsis arenosa*, *Arabidopsis thaliana* and *Cardamine parviflora* as outgroups. MAFFT v7.490 (Rozewicki et al., 2019) was used to align the sequences. IQ-tree 1.6.12 (Money and Whelan, 2012), using a ML model with 1,000 bootstrap replicates, constructed phylogenetic trees, and the best-fitting model was TVM + F + R3. The best-fitting model was TVM + F for the phylogenetic tree constructed within genera based on variation positions.

## Data Availability

The datasets presented in this study can be deposited in the NCBI repository, accession numbers can be found below: https://www.ncbi.nlm.nih.gov/genbank/, OM908760. https://www.ncbi.nlm.nih.gov/genbank/, OM908761. https://www.ncbi.nlm.nih.gov/genbank/, OM908762. https://www.ncbi.nlm.nih.gov/genbank/, OM914582. https://www.ncbi.nlm.nih.gov/genbank/, OK624380.

## Supplementary Material

Supplementary Figure S1 A comparison of the cp genomes among the 12 Tilia species. Gray arrows above the alignment indicate gene orientation. Genome regions are color-coded as exons, UTRs and non-coding sequences (CNSs). The vertical scale indicates the percentage of identity, ranging from 50% to 100%.
