# Supplementary figures and images for: Chloroplast Genomes of Genus Tilia: Comparative Genomics and Molecular Evolution
Source: Front Genet. 2022 Jul 8;13:925726. doi: 10.3389/fgene.2022.925726 (PMC9305825; doi:10.3389/fgene.2022.925726)

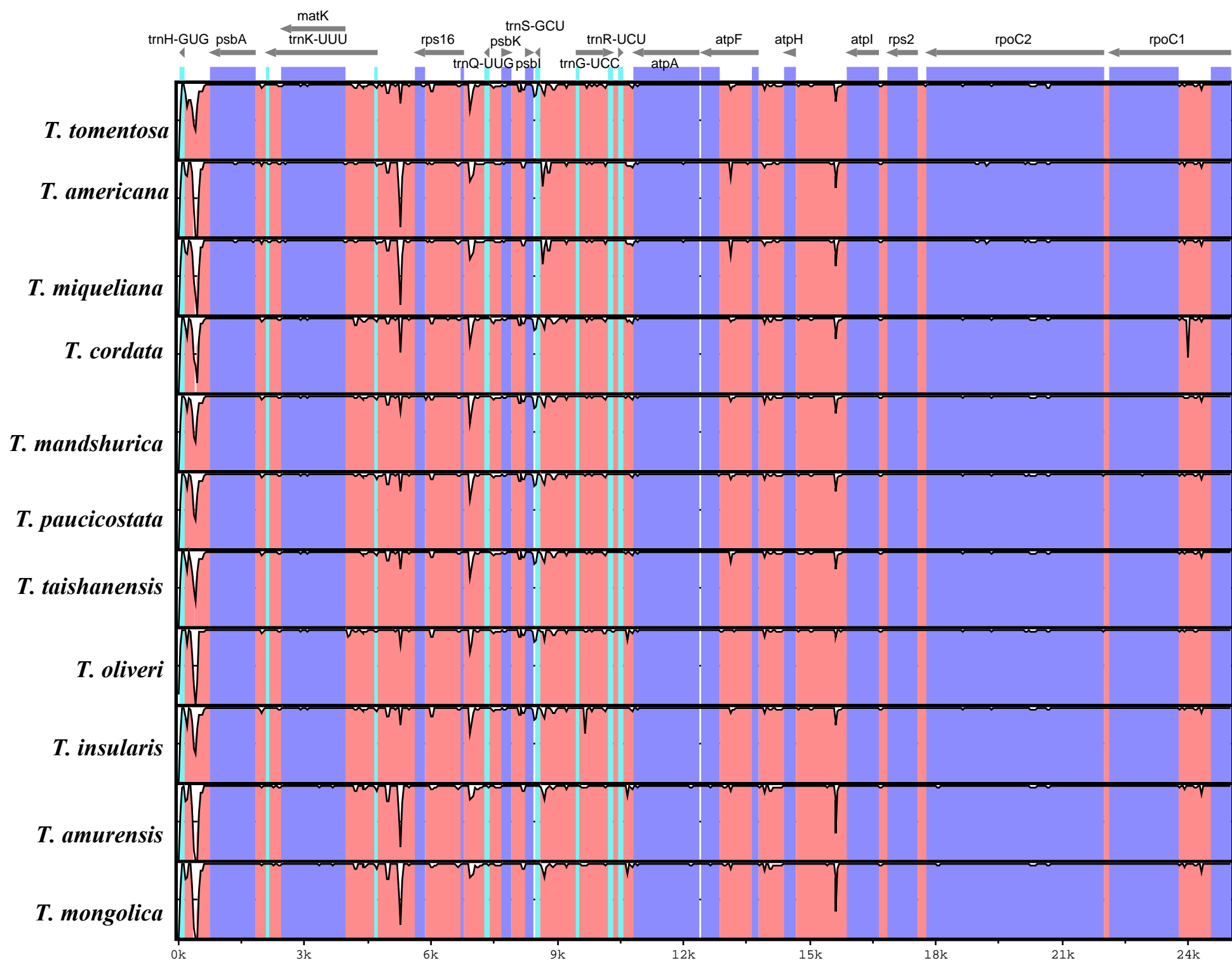

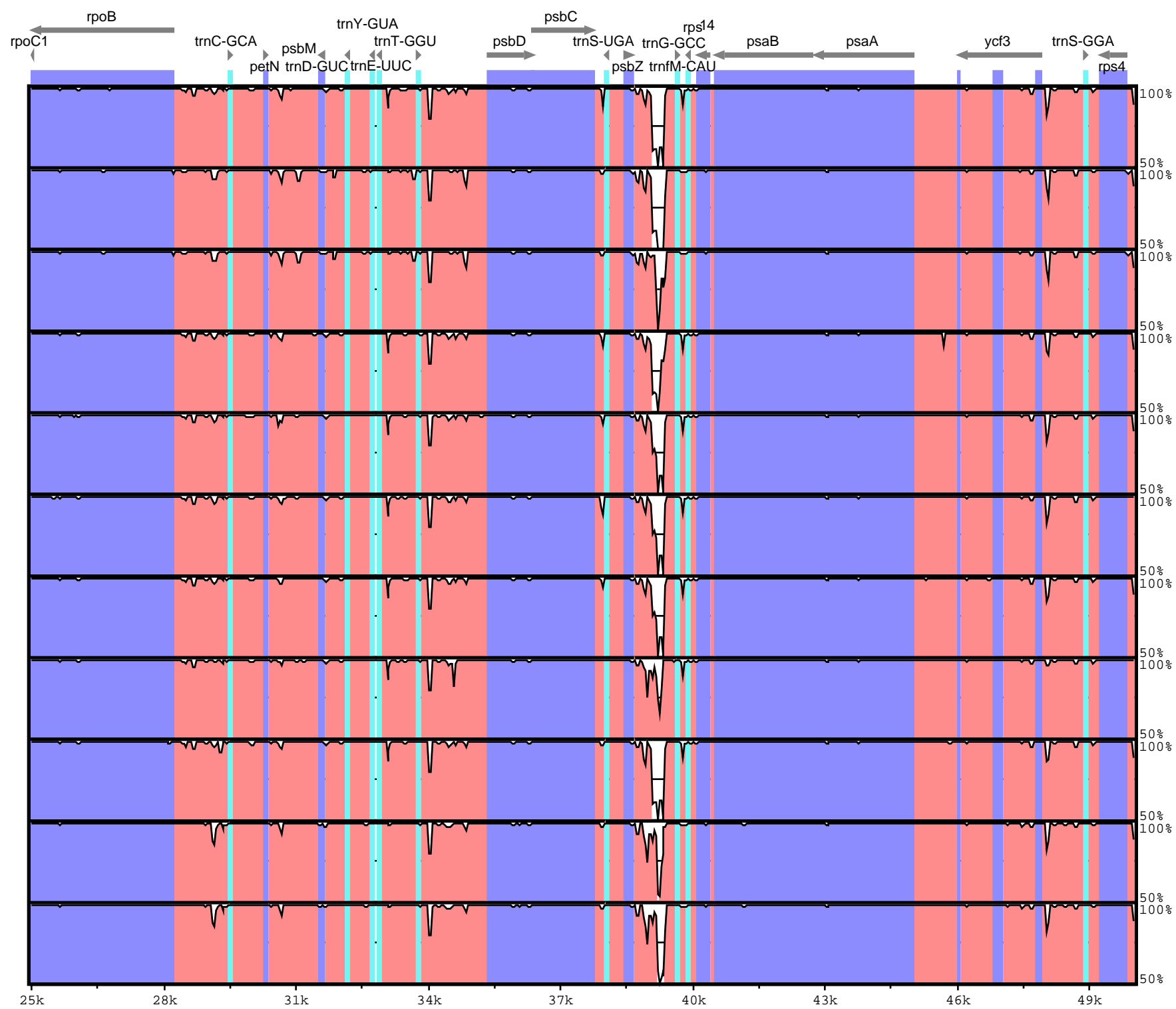

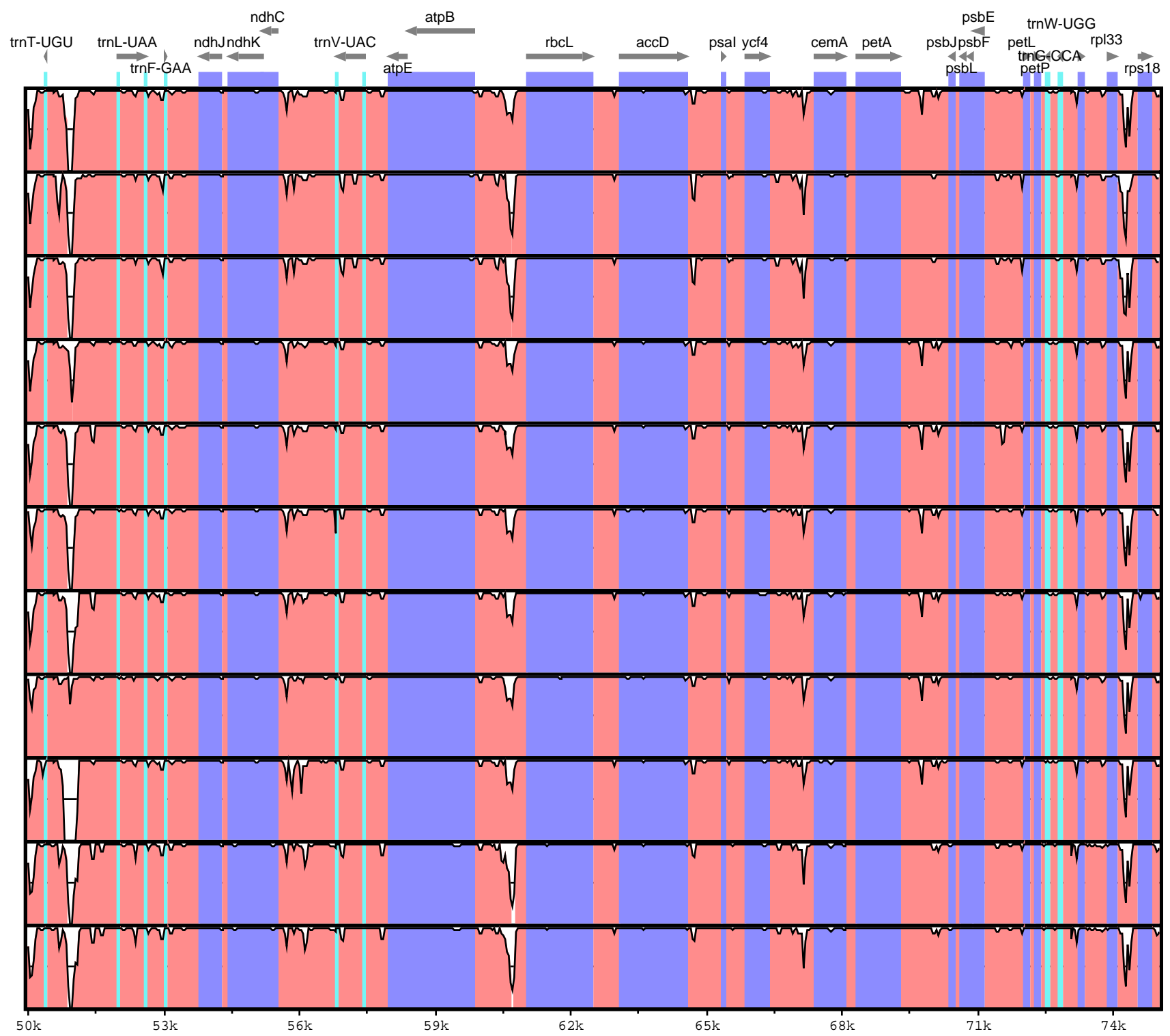

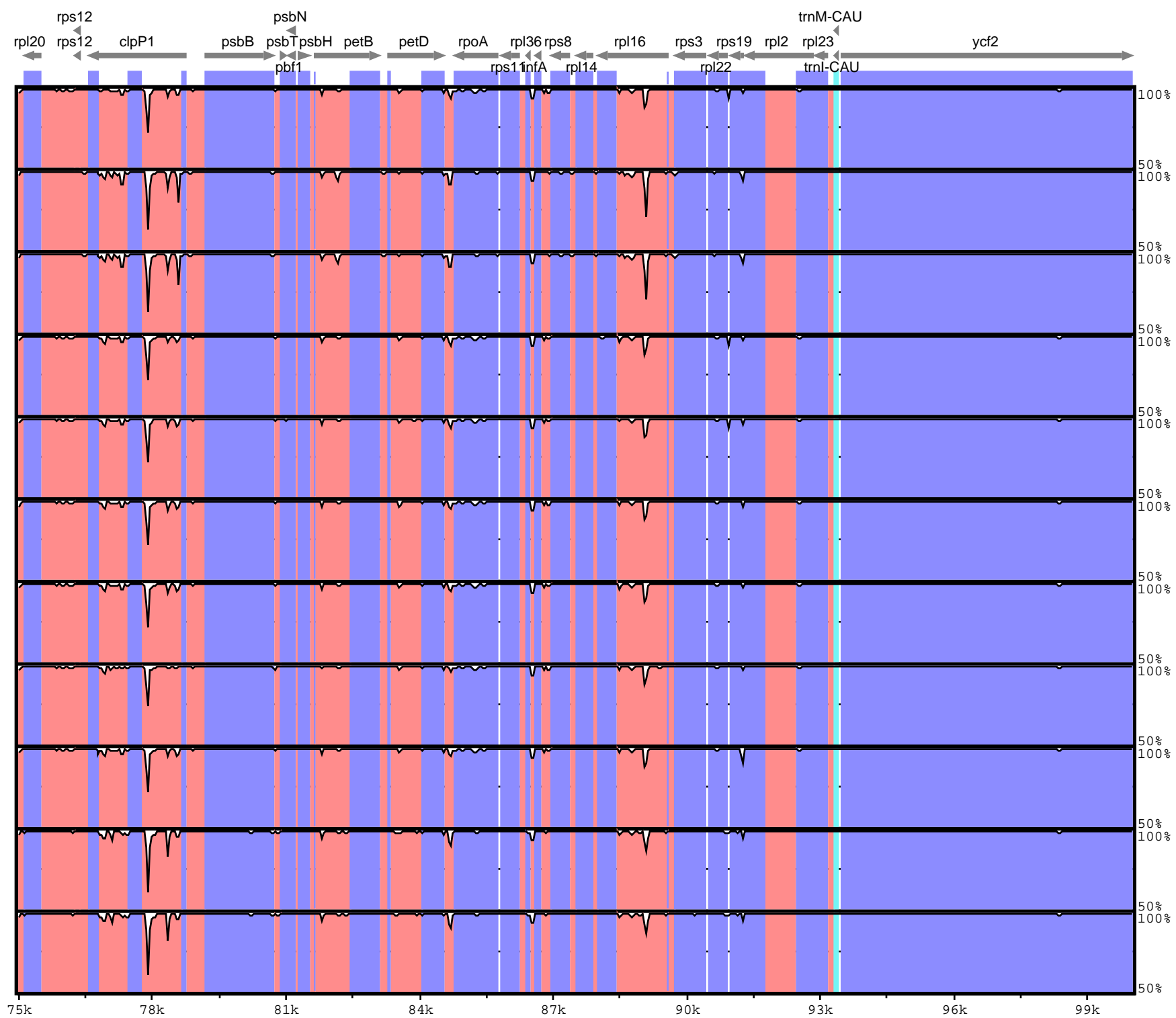

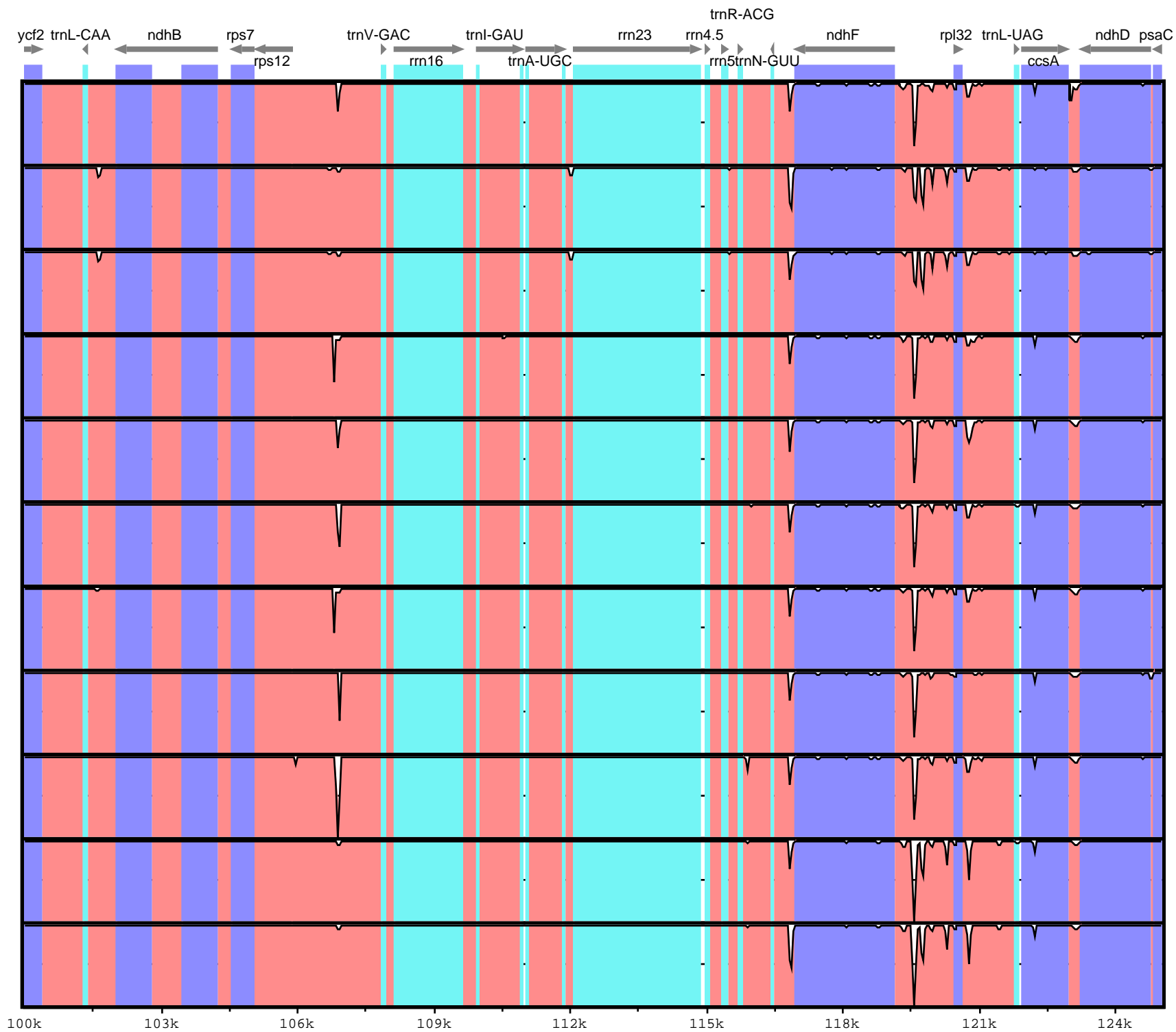

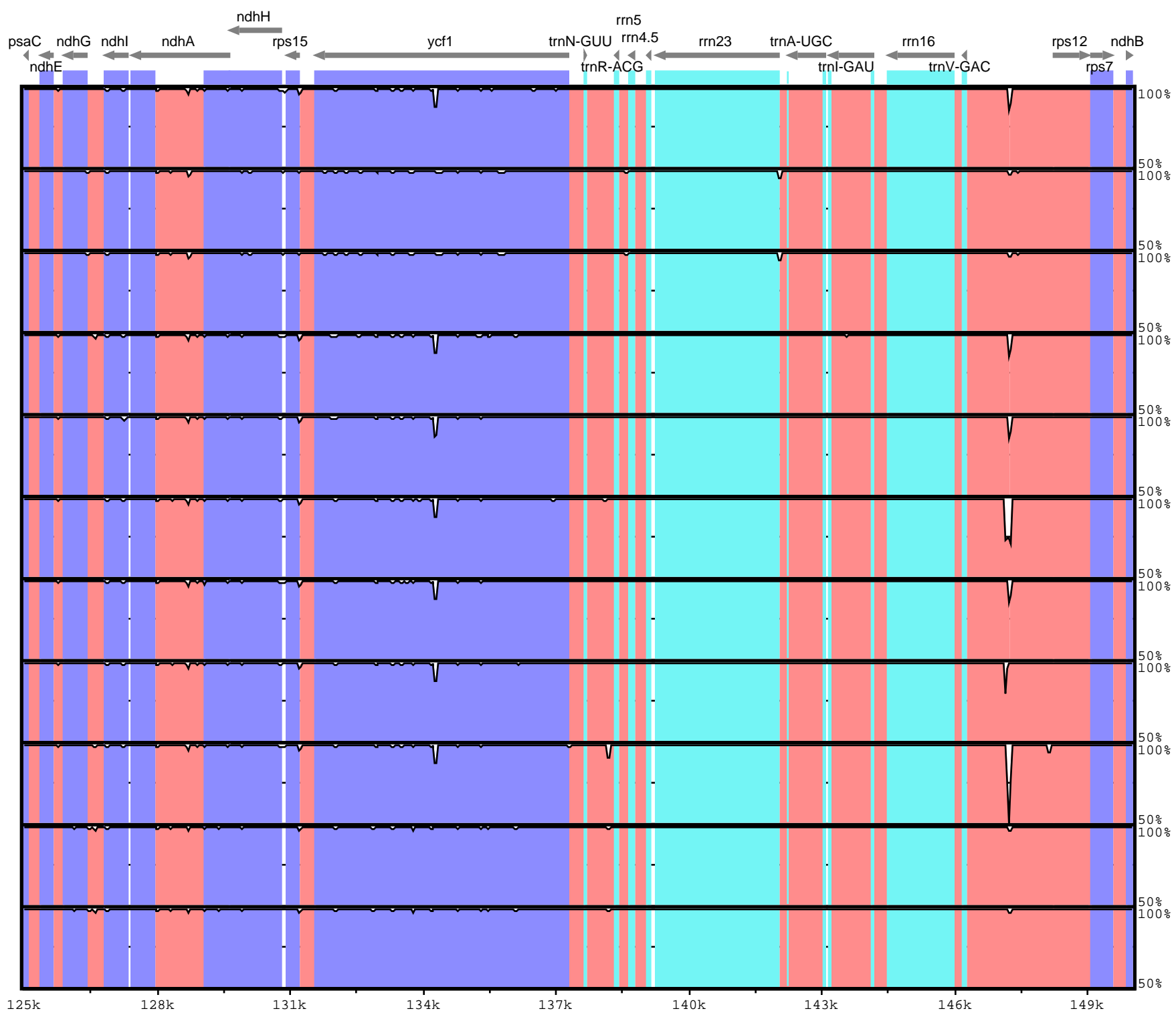

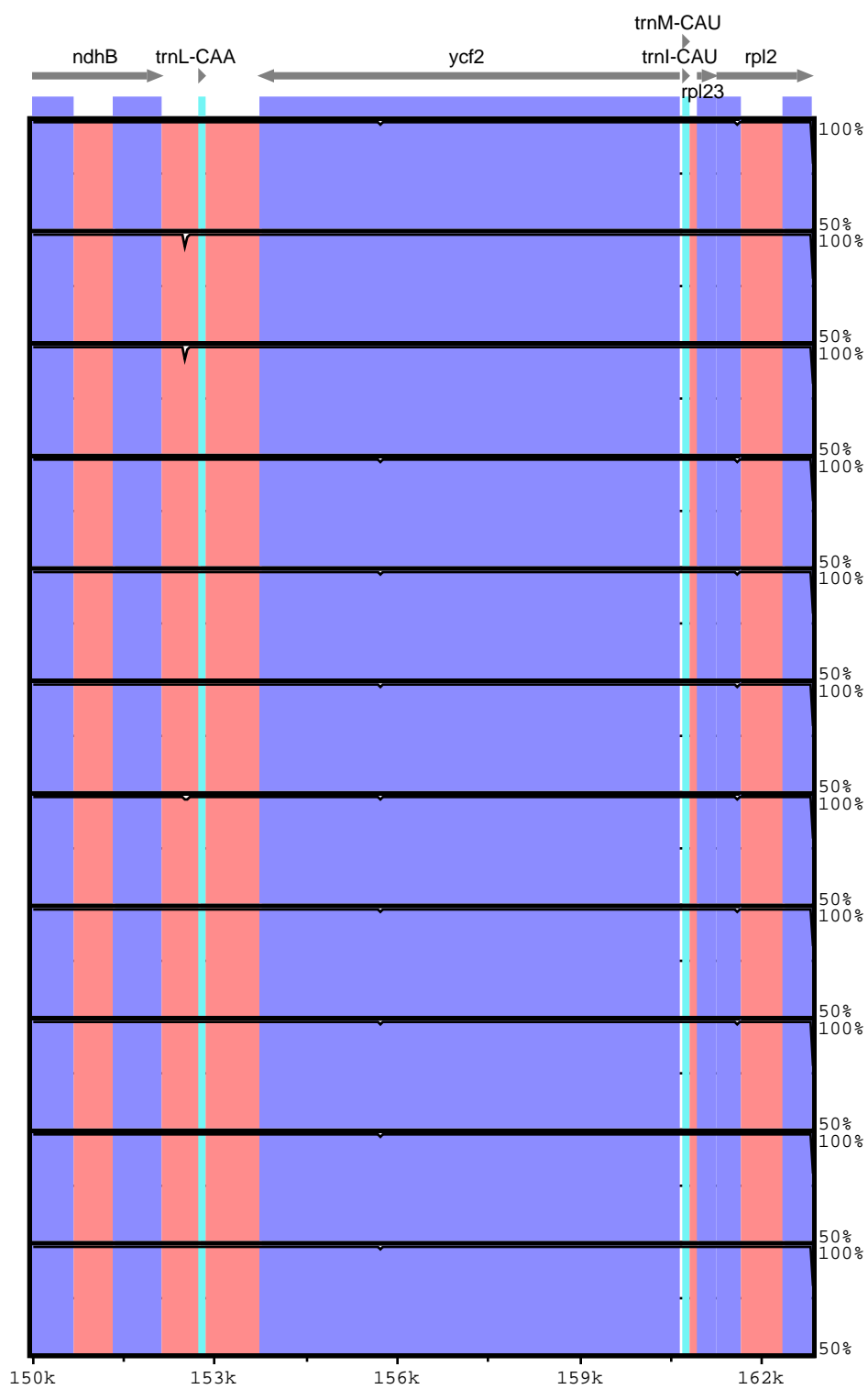

Supplement: Supplementary file 2 [file DataSheet1.PDF]
